# Isolated Transverse Colon Perforation During Corticosteroid Tapering in Quiescent Systemic Lupus Erythematosus: A Histopathology-Supported Diagnosis of Exclusion

**DOI:** 10.7759/cureus.112550

**Published:** 2026-07-12

**Authors:** Ridwan A Lawal, Nestor Malaga, Nida Asif, Fatimah Bello, Juliana Yang

**Affiliations:** 1 Internal Medicine, University of Texas Rio Grande Valley School of Medicine, Edinburg, USA; 2 Gastroenterology and Hepatology, University of Texas Rio Grande Valley School of Medicine, Edinburg, USA

**Keywords:** colonic perforation, corticosteroids, diagnosis of exclusion, histopathology, lupus enteritis, mesenteric vasculitis, pneumoperitoneum, prednisone, systemic lupus erythematosus, transverse colon

## Abstract

Gastrointestinal perforation in systemic lupus erythematosus (SLE) is uncommon but potentially fatal, and attribution is clinically consequential because perforation may reflect lupus enteritis with mesenteric vasculitis, infection, diverticular or stercoral disease, malignancy, inflammatory bowel disease (IBD), medication-related injury, or a combination of mechanisms. Corticosteroid-associated perforation is therefore best treated as a diagnosis of exclusion rather than as an isolated novel observation. We report a 62-year-old woman with SLE who developed sudden severe right-sided abdominal pain, nausea, vomiting, leukocytosis, lactic acidosis, and radiographic pneumoperitoneum while tapering prednisone from 60 mg/day to 10 mg/day over three months, with 10 mg/day documented at presentation in the available medication history. Computed tomography demonstrated free intraperitoneal air without radiologic diverticulitis or a clear alternative source. Emergent robotic-assisted laparoscopy was converted to open surgery because of extensive purulent contamination. Approximately 1.5 L of pus was evacuated, and a solitary mid-transverse colon perforation was treated with segmental colectomy and primary anastomosis. Gross pathology showed an irregular ulcer with a transmural defect and no mass or diverticulum. Histopathology demonstrated a full-thickness perforated ulcer with acute-on-chronic inflammation and granulation tissue, without vasculitis, thrombosis, ischemic necrosis, granulomas, IBD, or malignancy. The postoperative course was uncomplicated, with the return of bowel function by postoperative day 4 and discharge on day 6. This case is unusual because SLE-associated intestinal perforation is more commonly described in the context of active lupus mesenteric vasculitis or other identifiable etiologies. The report emphasizes a structured exclusion-based diagnostic approach, early source control, and postoperative reassessment of glucocorticoid exposure and steroid-sparing therapy.

## Introduction

Systemic lupus erythematosus (SLE) is a chronic multisystem autoimmune disease with marked geographic, racial, ethnic, and sex-related variation. A recent global epidemiologic analysis estimated a worldwide SLE incidence of approximately 5.14 per 100,000 person-years, with a substantially higher incidence among women than men [1].

Modern SLE management emphasizes disease control while minimizing cumulative glucocorticoid exposure because long-term steroids contribute to organ damage, infection, metabolic complications, and medication-related gastrointestinal injury [2].

Gastrointestinal involvement is reported in approximately 40-60% of patients with SLE; however, abdominal symptoms may arise from medications, infection, pancreatobiliary or peptic disease, thromboembolism, diverticular disease, and other non-SLE causes [3,4]. In corticosteroid-treated patients, distinguishing active lupus-related disease from medication-associated injury is particularly difficult because competing mechanisms can overlap.

Lupus enteritis, also called lupus mesenteric vasculitis, is an important lupus-specific abdominal emergency. Systematic review and cohort data associate it with abdominal pain, hypocomplementemia, features of active systemic disease, ascites, bowel wall abnormalities, and mesenteric inflammatory changes; histologic confirmation of vasculitis is uncommon [5,6].

Typical radiologic features of lupus enteritis include bowel-wall thickening or edema, target or double-halo enhancement, mesenteric vessel engorgement, ascites, and mesenteric fat stranding. Their absence does not exclude lupus enteritis, but active vasculitic perforation becomes less likely when operative and histologic findings do not support vasculitis [7].

True intestinal perforation in SLE is rare. A 2023 systematic review identified 18 published cases between 1984 and 2022; 94.4% had high SLE disease activity, the rectum was the most frequent site, and lupus mesenteric vasculitis accounted for two-thirds of cases [8]. Earlier reports likewise emphasized active disease and high mortality [9].

Isolated transverse colon perforation during clinical quiescence and steroid tapering is rarely described. This report presents a solitary mid-transverse colon perforation in a patient with clinically quiescent SLE after recent high-dose prednisone exposure and addresses the clinical gap through structured differentiation between lupus enteritis and steroid-associated injury. Exclusion of competing etiologies can support steroid-associated injury as the most plausible explanation, but cannot prove direct causation.

This article was previously presented as a meeting abstract at the 2025 American College of Gastroenterology (ACG) Annual Scientific Meeting on October 26, 2025.

## Case presentation

A 62-year-old woman with a known history of SLE presented to the emergency department with sudden, severe abdominal pain, most prominent in the right upper and right lower quadrants, accompanied by nausea and vomiting. She had recently received high-dose prednisone for SLE, tapered from 60 mg/day to 10 mg/day over three months. The available medication history indicated that she was still taking prednisone 10 mg/day at the time of presentation. She denied nonsteroidal anti-inflammatory drug use and had no known history of diverticular disease, inflammatory bowel disease (IBD), colorectal malignancy, or previous colonic surgery.

Within the available source record, no nephritis, serositis, rash, active arthritis, leukopenia, thrombocytopenia, or other clear flare feature was documented at presentation. Complement levels, anti-double-stranded DNA antibody titers, erythrocyte sedimentation rate, C-reactive protein, urinalysis, renal activity markers, the exact platelet count (although it was reported as normal), previous colonoscopy or colorectal cancer screening history, and a full non-prednisone medication list were not available; therefore, clinical quiescence was inferred from the provided clinical record rather than confirmed serologically.

**Figure 1:**
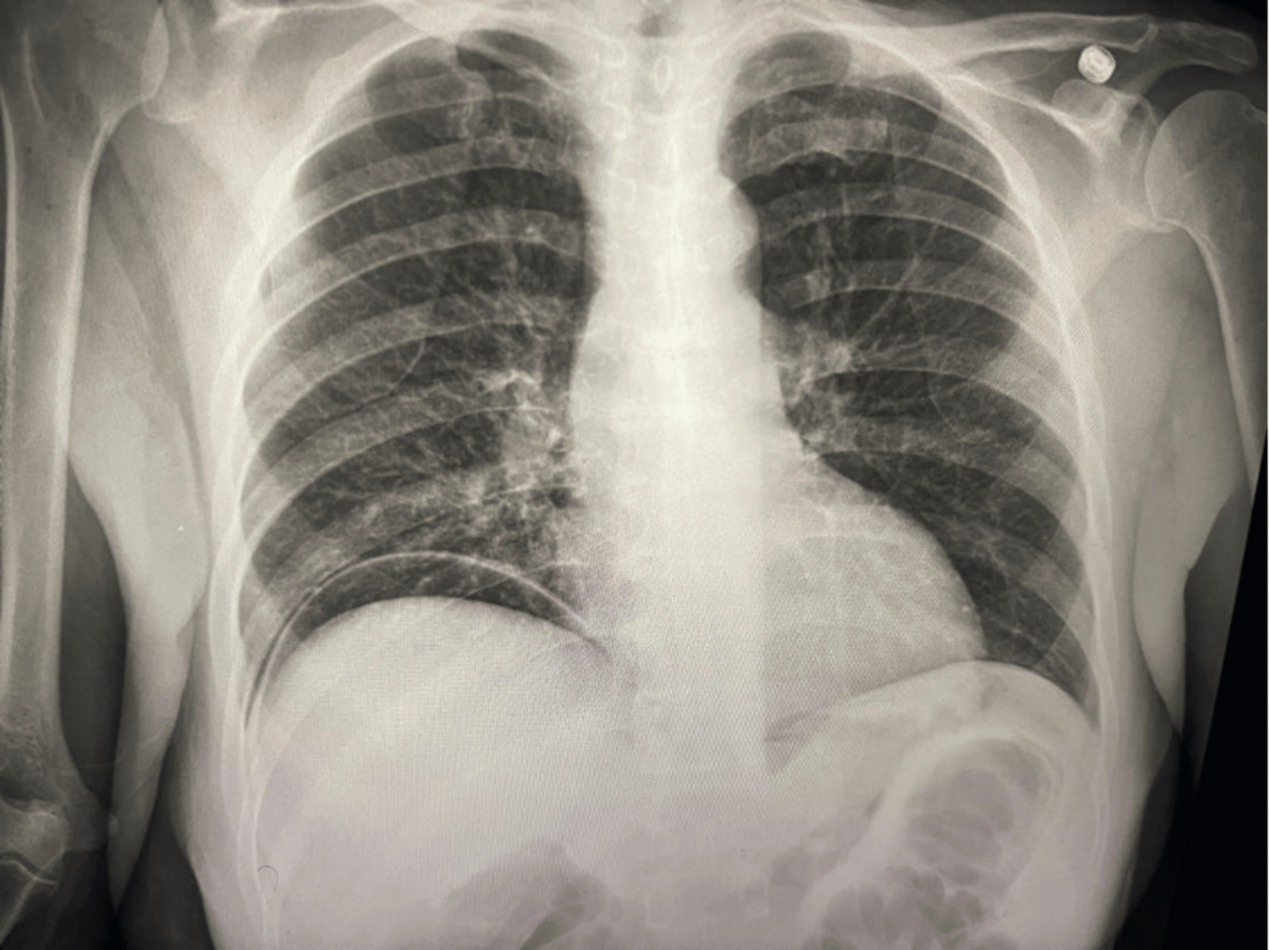
Upright chest radiograph demonstrating free subdiaphragmatic air consistent with pneumoperitoneum.

**Figure 2:**
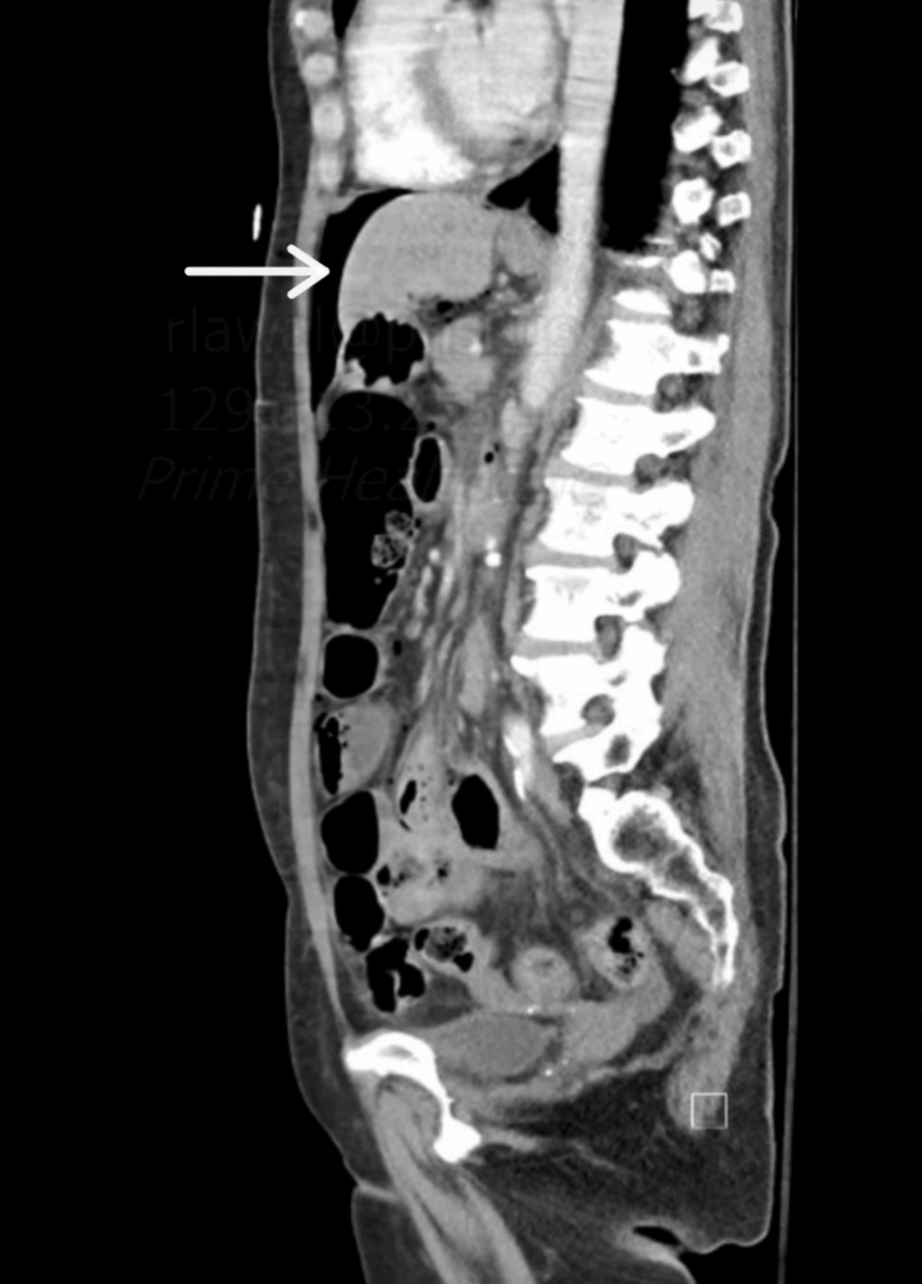
Sagittal CT image demonstrating pneumoperitoneum/free intraperitoneal air in the setting of hollow viscus perforation (arrow). CT: computed tomography

On examination, she was afebrile but appeared uncomfortable. Abdominal examination demonstrated diffuse tenderness, greatest in the right upper abdomen, with guarding. The cardiopulmonary examination was unremarkable. Initial laboratory testing showed leukocytosis, anemia, and elevated lactate, while the basic metabolic panel was reported as normal. An upright chest radiograph demonstrated free subdiaphragmatic air (Figure 1), and computed tomography of the abdomen confirmed pneumoperitoneum without a clear source of perforation or evidence of diverticulitis (Table 1 and Figure 2).

**Table 1:** Clinical, laboratory, and imaging profile at presentation SLE: systemic lupus erythematosus; CT: computed tomography

Domain	Finding	Diagnostic relevance
Age/sex	62-year-old woman	Older age and female sex are typical of many reported SLE gastrointestinal complications, but neither establishes etiology.
SLE status	Known SLE; no documented nephritis, serositis, rash, active arthritis, leukopenia, thrombocytopenia, or other clear flare features at presentation in the provided record	Clinically favored quiescent SLE rather than active lupus enteritis.
Steroid exposure	Prednisone tapered from 60 mg/day to 10 mg/day over three months; 10 mg/day documented at presentation	
Symptoms	Sudden severe right-sided abdominal pain with nausea and vomiting	Consistent with hollow viscus perforation but not specific for the cause.
Examination	Diffuse abdominal tenderness, greatest in the right upper abdomen, with guarding; afebrile	Steroids can blunt fever and peritoneal signs; so, the absence of fever did not reassure.
White blood cell count	14.2 x 10^9^/L	Leukocytosis supported acute an inflammatory or infectious intra-abdominal process.
Hemoglobin	10.3 g/dL	Anemia was present; platelet count was 264 x 10^3^/µ L
Lactate	3.8 mmol/L	Elevated lactate supported physiologic severity and need for urgent source control.
Basic metabolic panel	Reported as normal	
Radiography	Free subdiaphragmatic air	Confirmed pneumoperitoneum.
CT abdomen	Free intraperitoneal air; no clear source and no diverticulitis reported	Did not demonstrate classic lupus enteritis features or diverticulitis.

Diagnostic assessment

The central diagnostic problem was attribution of a solitary colonic perforation in a patient whose underlying disease and therapy could both plausibly injure the bowel. Because corticosteroid exposure is associated with gastrointestinal injury and impaired healing, while SLE can cause mesenteric vasculitis, the working differential diagnosis included lupus enteritis/lupus mesenteric vasculitis, steroid-associated ulceration and impaired healing, diverticular perforation, stercoral ulceration, infectious colitis including cytomegalovirus (CMV) in the immunosuppressed host, IBD, malignancy, ischemic colitis, and nonsteroidal anti-inflammatory drug (NSAID)-related injury (Table 2).

**Table 2:** Differential diagnosis and exclusion-based reasoning NSAID: nonsteroidal anti-inflammatory drugs; IBD: inflammatory bowel disease; CMV: cytomegalovirus; CT: computed tomography; SLE: systemic lupus erythematosus

Potential etiology	Expected supportive evidence	Patient-specific findings	Interpretation
Lupus enteritis or mesenteric vasculitis	Active SLE features, hypocomplementemia, leukopenia, ascites, bowel-wall edema, target sign, mesenteric vessel engorgement, vasculitis, or thrombosis on pathology	SLE appeared clinically quiescent; CT did not report classic enteritis features; pathology showed no vasculitis, thrombosis, or ischemic necrosis	Unlikely based on available clinical, radiologic, operative, and histologic data
Steroid-associated perforated ulcer	Recent or prolonged high-dose corticosteroid exposure, muted symptoms, focal ulceration, impaired healing, and absence of an alternative structural cause	Prednisone 60 mg/day tapered to 10 mg/day over three months; afebrile presentation; solitary perforated ulcer with acute-on-chronic inflammation and granulation tissue; no mass or diverticulum	Favored diagnosis after exclusion of competing etiologies
Diverticular perforation	Diverticula at or near perforation, pericolic inflammatory changes, abscess, typical sigmoid involvement	No diverticulum identified grossly; CT did not report diverticulitis; the site was mid-transverse colon	Not supported
Stercoral perforation	Fecaloma, pressure necrosis, chronic constipation, antimesenteric ulceration, rectosigmoid predominance	No fecaloma or stercoral ulcer features were reported; perforation was in the mid-transverse colon	Less likely
Infectious colitis, including cytomegalovirus	Viral inclusions, ulceration in immunosuppression, positive stains or serology when performed	No viral cytopathic effect was reported on histology; no infectious colitis diagnosis was provided	Less likely, though CMV immunostaining was not performed
Inflammatory bowel disease	Chronic architectural distortion, granulomas in Crohn's disease, multifocal ulcers, compatible history	No previous IBD history; pathology showed no IBD or granulomas	Not supported
Malignancy	Mass lesion, dysplasia, invasive tumor, obstructive symptoms	Gross examination showed no mass; histology showed no malignancy	Excluded pathologically
NSAID-related ulceration	NSAID exposure, diaphragm-like strictures, ulceration	Patient denied NSAID use	Less likely

Treatment and follow-up

The patient was treated initially with broad-spectrum intravenous antibiotics and emergent surgical consultation. She underwent robotic-assisted diagnostic laparoscopy, which was converted to an open operation because of extensive purulent contamination. Approximately 1.5 L of pus was evacuated. Exploration identified a solitary perforation in the mid-transverse colon. Segmental colectomy with primary anastomosis was performed. The source packet did not provide enough operative detail to verify the decision-making factors for primary anastomosis, including hemodynamic stability, vasopressor requirement, bowel viability, and the rationale for avoiding diversion.

**Figure 3:**
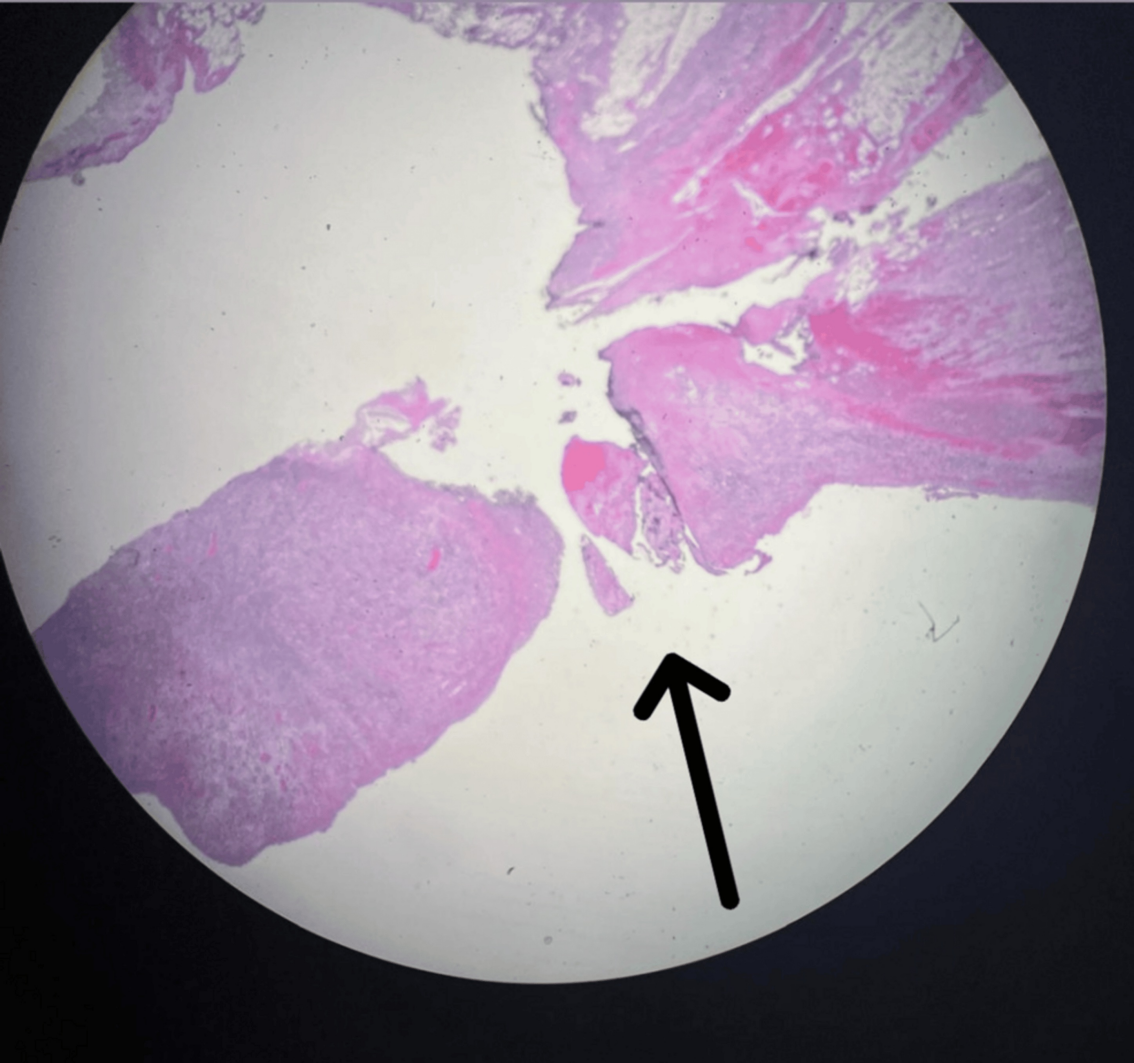
Low-power histopathology image from the resected specimen showing ulcerated/perforated bowel wall with acute-on-chronic inflammation (arrow). The magnification, higher-power image, and additional vessel-focused sections were unavailable.

Gross examination of the resected specimen showed an irregular ulcer with a transmural defect, without diverticulum or mass. Histopathologic examination demonstrated a full-thickness perforated ulcer with acute-on-chronic inflammation and granulation tissue (Figure 3). The available pathology description did not further specify the layer-by-layer distribution of inflammation, detailed mesenteric or submucosal vessel morphology, whether microvascular thrombosis was separately assessed beyond the general report of no thrombosis, or subtle ischemic changes beyond the reported absence of ischemic necrosis. No vasculitis, thrombosis, ischemic necrosis, granulomatous inflammation, IBD, or malignancy was reported. CMV immunostaining was not performed. The postoperative course was uncomplicated: abdominal pain improved, bowel function returned by postoperative day 4, and she was discharged on postoperative day 6 with outpatient surgical and rheumatology follow-up and a revised immunosuppressive plan intended to reduce unnecessary glucocorticoid exposure while maintaining SLE control.

## Discussion

The available evidence does not establish corticosteroids as the direct cause of perforation; steroid-associated injury remains an exclusion-based interpretation. The significance of this case lies in the convergence of an unusual location, recent high-dose prednisone exposure, clinical quiescence of SLE within the available record, absence of diverticular disease or mass, lack of classic lupus enteritis imaging, and histology that did not support vasculitis, thrombosis, ischemic necrosis, granulomas, IBD, or malignancy. Together, these findings make steroid-associated injury the most plausible explanation after exclusion rather than a proven causal diagnosis.

An association with corticosteroid exposure is biologically plausible, but mechanistic plausibility alone does not establish causation. Corticosteroids reduce inflammatory signaling, impair fibroblast proliferation and collagen deposition, blunt macrophage and neutrophil responses, decrease prostaglandin-mediated mucosal protection, and delay ulcer healing. Clinically, this may both predispose to ulcer progression and obscure early peritoneal symptoms. In a systematic review and meta-analysis of randomized trials, corticosteroids increased the risk of gastrointestinal bleeding or perforation by approximately 40%, while a classic surgical series emphasized that high-dose glucocorticoids can reduce the clinical expression of peritonitis and delay recognition of perforation [10,11].

Initial management appropriately prioritized sepsis prevention and source control rather than prolonged etiologic debate. Current intra-abdominal infection pathways emphasize early recognition, physiologic resuscitation, prompt initiation of broad-spectrum antimicrobial therapy, and definitive source control; in this patient, pneumoperitoneum, guarding, leukocytosis, elevated lactate, and operative contamination all warranted urgent surgical intervention [12].

The most important postoperative question was whether the perforation represented active lupus enteritis requiring escalation of immunosuppression or steroid-associated injury requiring steroid minimization. Several features argued against lupus enteritis: the bowel lesion was solitary and colonic rather than diffuse or small-bowel predominant; CT did not report target sign, diffuse bowel-wall edema, ascites, or mesenteric vessel engorgement; the patient lacked documented systemic flare features; and the resected colon showed no vascular inflammation, thrombosis, or ischemic necrosis. These findings support steroid-associated perforated ulcer as the most plausible diagnosis after systematic exclusion, although serologic markers of SLE activity and CMV immunostaining were unavailable.

Published cases of SLE perforation illustrate why this distinction matters. Reports include lupus-associated ileal perforation, CMV-associated colonic perforation, stercoral perforation, and steroid-induced colonic perforation, demonstrating that patients with SLE can perforate through autoimmune, infectious, mechanical, and treatment-related pathways, each requiring different postoperative decisions [13-15].

The practical lesson is that a patient with SLE and pneumoperitoneum should be managed surgically first and categorized etiologically second. After source control, the pathology report should be reviewed with rheumatology and surgery, medication exposure should be reassessed, and steroid-sparing SLE control should be considered collaboratively within accepted classification and management frameworks [16,17].

This case has limitations. Complement levels, anti-double-stranded DNA antibody titers, urinalysis, erythrocyte sedimentation rate, C-reactive protein, detailed platelet count, renal activity markers, full non-prednisone medication history, previous colonoscopy or colorectal cancer screening history, CMV immunostaining, original histopathology stain and magnification, and a higher-power histopathology image were not provided in the source packet. The available pathology description also lacked a layer-specific account of inflammation, detailed mesenteric and submucosal vessel morphology, a separate assessment of microvascular thrombosis, and characterization of subtle ischemic changes. These missing data mean that clinical quiescence was not serologically confirmed, CMV could not be completely excluded, and corticosteroid-associated injury remains an inferential association rather than a proven cause. Nonetheless, the operative and histopathologic findings exclude several competing etiologies and support steroid-associated injury as the most plausible diagnosis after exclusion.

## Conclusions

Isolated perforation of the transverse colon during a corticosteroid tapering in clinically quiescent SLE is rare and diagnostically challenging. The value of this case lies in the exclusion-based demonstration that active lupus enteritis, diverticular disease, IBD, malignancy, and other obvious structural causes were not supported by the available clinical, imaging, operative, and histopathologic data. Because serologic markers of SLE activity and CMV immunostaining were unavailable, clinical quiescence was not serologically confirmed, and CMV infection could not be completely excluded. In patients with SLE presenting with an acute abdomen, clinicians should maintain a high index of suspicion because corticosteroids may blunt symptoms, proceed rapidly to imaging and surgical source control when pneumoperitoneum is present, and use histopathology to guide postoperative immunosuppressive strategy. When vasculitis is absent, and a focal perforated ulcer is present after high-dose steroid exposure, steroid-associated injury should be considered the most plausible explanation after exclusion, rather than a proven causal diagnosis. Postoperative glucocorticoid-sparing management should be considered in collaboration with rheumatology whenever clinically feasible.
